# Electrochemical Improvement of the MWCNT/Al Electrodes for Supercapacitors

**DOI:** 10.3390/ma14247612

**Published:** 2021-12-10

**Authors:** Arkady N. Redkin, Alena A. Mitina, Eugene E. Yakimov, Evgeny N. Kabachkov

**Affiliations:** 1 Institute of Microelectronics Technology and High-Purity Materials, Russian Academy of Science (IMT RAS), Moscow District, 6 Academician Ossipyan Str., 142432 Chernogolovka, Russia; alena@iptm.ru (A.A.M.); yak@iptm.ru (E.E.Y.); 2 Institute of Problems of Chemical Physics of Russian Academy of Sciences (IPCP RAS), Moscow Region, 1 Academician Semenov Av., 142432 Chernogolovka, Russia; en.kabachkov@gmail.com; 3 Chernogolovka Scientific Center, Russian Academy of Sciences, Moscow Region, 142432 Chernogolovka, Russia

**Keywords:** CVD, supercapacitors, carbon nanotubes on aluminum foil, electrochemical oxidation

## Abstract

An original technique of chemical deposition (CVD) by catalytic pyrolysis of ethanol vapor was used to directly grow multiwall carbon nanotubes (MWCNTs) layers on aluminum foil. The grown nanotubes had excellent adhesion and direct electrical contact to the aluminum substrate. This material was perfect for use in electrochemical supercapacitors. In this work, the possibility of a significant increase in the specific capacity of MWCNTs by simple electrochemical oxidation was investigated. The optimal conditions for improving the characteristics of the MWCNT/Al electrodes were found. Electrochemical treatment of MWCNT/Al electrodes in a 0.005 M Na_2_SO_4_ solution at a potential of 4–5 V for 20–30 min increased the specific capacity of MWCNTs from 30 F/g to 140 F/g. The properties of modified nanotubes were investigated by X-ray photoelectron spectroscopy, cyclic voltammetry (CV), and impedance spectroscopy. A significant increase in the concentration of oxygen-containing functional groups on the surface of MWCNTs was found as a result of electrochemical oxidation. The modified MWCNT/Al electrodes maintained excellent stability to multiple charge–discharge cycles. After 20,000 CVs, the capacity loss was less than 5%. Thus, the results obtained significantly expanded the possibilities of using MWCNT/Al composite materials obtained by the method of direct deposition of carbon nanotubes on aluminum foil as electrodes for supercapacitors.

## 1. Introduction

Numerous useful properties of carbon nanomaterials, such as a large specific surface area, low specific gravity, excellent chemical resistance, low cost and abundant supply of starting materials for their production, make them very attractive for widespread use in various fields of practical application [1,2,3]. Among them, carbon nanomaterials are often applied to promising energy sources [4,5,6]. They are of particular interest for developing electric double layer capacitors (EDLCs) or supercapacitors, as their characteristics directly depend on the surface area of the working electrodes increased by carbon nanomaterials. In this regard, carbon nanotubes (CNTs) are of constant interest in this field of application, since they possess all the properties required (e.g., high specific surface area, low electrical resistance and chemical stability) for use in supercapacitors [7,8,9]. At the same time, the value of the specific capacity of the raw (untreated) CNTs as an active material of supercapacitors does not exceed several tens of F/g when they are an active material for supercapacitors [10,11,12]. This is not enough to effectively compete with modern nanomaterials with hundreds of F/g [9]. There have been methods reported [13,14,15,16] for achieving significant improvements in the characteristics of CNTs involving surface functionalization. The most common method for activating the CNT surface is the treatment of materials with strong oxidants. More than 40% increase in capacity was observed after treatment of carbon fabrics with concentrated nitric acid [13]. Frackowiak et al. [14] observed that the values of specific capacitance varied from 4 to 135 F/g, depending on the type of nanotubes or/and their posttreatments. Kang et al. [15] obtained MWCNTs with specific capacity of 147 F/g after oxidation with chlorate and thermal deoxygenation in air. In the work of Frackowiak et al. [16], MWCNTs were activated with KOH at 800 °C under argon flow, resulting in a nearly 7-fold increase in their specific capacity (from 15 to 90 F/g). However, this method of nanotube activation has a number of significant drawbacks. High temperature processing with strong oxidants is dangerous and requires strict precautions. Aggressive reagents and wash water must be disposed of after CNT processing. Activated nanotubes acquire hydrophilic properties and are difficult to separate from the solution. When electrodes are made of bulk materials, functionalization of the nanotube surface can lead to an increase of the electrical resistance in the active layer and in the contact with the current collector.

At present, the processes of direct growth of CNTs on metal substrates are being developed. Such composite materials can be directly used as supercapacitor electrodes [17,18,19]. Methods for growing CNTs on an aluminum substrate are of great interest, as aluminum is the most suitable inexpensive material for electrodes due to its properties (low specific gravity −2.7 g/cm^3^, low specific electrical resistivity −2.7 × 10^−8^ Ohm∙m, high plasticity, etc., http://www.matweb.com, accessed on 10 September 2021). There are serious limitations associated with direct deposition of CNTs on aluminum including a lack of catalytic properties and a relatively low melting point (660 °C) of this metal. Nevertheless, several authors have demonstrated successful results on the deposition of CNTs on aluminum substrates and the use of the obtained materials as supercapacitor electrodes [20,21,22,23,24,25]. 

Specific capacitance is one of the most important characteristics of a supercapacitor electrode material for energy storage. As mentioned above, specific capacity of pristine nanotubes can be significantly increased by oxidation. The problem of increasing the specific capacity is also relevant for CNTs on aluminum substrates. Obviously, methods of improving performance by treating with concentrated acids and alkalis under harsh conditions are not applicable to this category of materials. In this case, the method of electrochemical modification of CNTs is more suitable. The method consists of anodic oxidation of nanotubes to form oxygen-containing functional groups on their surface [26,27,28,29]. During electrochemical oxidation, the surface of nanotubes can be etched with the formation of pores and an increase in the specific surface area [29,30]. 

The advantages of electrochemical oxidation are the absence of aggressive reagents, control of the degree of modification, and the possibility of improving the characteristics of ready-made electrodes. The results of electrochemical oxidation may depend on both the processing conditions and on the quality of CNTs (the degree of their defectiveness). Earlier, a simple and reliable method was developed for the deposition of highly defective MWCNTs on aluminum foil by pyrolysis of ethanol vapors. The material obtained was then successfully applied as supercapacitor electrodes by us [31]. In contrast, the present work will investigate the possibility of improving the characteristics of such MWCNT/Al electrodes using the method of electrochemical modification.

## 2. Materials and Methods

A 50 μm thick aluminium foil was used as a substrate. The following steps were carried out to provide catalytic properties to the foil. It was cleaned with isopropyl alcohol and distilled water and cut into 1.5 cm × 3 cm pieces. The prepared aluminum strips were sonicated for 5 min on each side in a suspension of 40 mL of 20 wt% Ni(NO_3_)_2_ solution and 45 mg of corundum of 28 μm. Then, they were washed with distilled water. Further mild oxidation of the samples carried out in a 20 wt% aqueous solution of Ni(NO_3_)_2_ for 20 h. Then, they were washed several times with deionized water to remove residual Ni(NO_3_)_2_ and dried in air. MWCNTs were deposited on the prepared aluminum foil by the pyrolysis of ethanol vapor on a nickel catalyst as described previously [31,32,33]. A home-made setup was used with a flow-type silica reactor, an external electric furnace, and peristaltic pump to control the flow of liquid ethanol [31]. No gaseous reagents were used. MWCNTs were deposited at atmospheric pressure at temperature 600 °C for 1 h. Alcohol consumption was 6–7 mL/h. The masses of the deposited MWCNTs were calculated from the difference in the masses of the substrates before and after synthesis. 

A two-electrode cell was used for the electrochemical oxidation of the samples under study. The MWCNT/Al strip served as an anode, the potential of which varied from 2 to 7 V. A platinum wire was used as a counter electrode. An aqueous solution of sodium sulfate of various concentrations was used as the electrolyte. The entire process was carried out in several steps with the intermediate measurement of the characteristics of the sample. The total oxidation time was usually 1 h. 

Electrochemical testings of the initial and modified samples (cyclic voltammetry, galvanostatic charge–discharge tests, and impedance spectroscopy) were carried out in a three-electrode cell. A saturated calomel electrode (SCE; warning: care must be exercised in handling the SCE owing to health hazard of mercury.) and a platinum wire were, respectively, used as a reference electrode and a counter electrode. Strips of aluminium foil (0.75 cm × 3 cm) coated on both sides with a layer of MWCNTs were used as working electrodes. A 0.5 M aqueous solution of Na_2_SO_4_ was used as the electrolyte. Electrochemical impedance experiments were conducted in the presence of 0.5 M aqueous Na_2_SO_4_ solution at a DC potential of 0 V, superimposed by an AC potential of 20 mV peak-to-peak amplitude over a frequency range of 50 kHz to 10 mHz. All potentiostatic and impedimetric experiments were carried out using a P-40X potentiostat (Electrochemical Instruments, Moscow, Russia).

Morphology of the samples was examined using a JSM 6490 scanning electron microscope. X-ray photoelectron spectroscopy was conducted using a Specs PHOIBOS 150 MCD9 spectrometer for chemical analysis (SPECS Gmbh, Berlin, Germany) and an X-ray tube with a magnesium anode (Mg Kα—radiation 1253.6 eV). Peak fitting of the spectra was performed with the Origin software.

## 3. Results and Discussions

**Figure 1:**
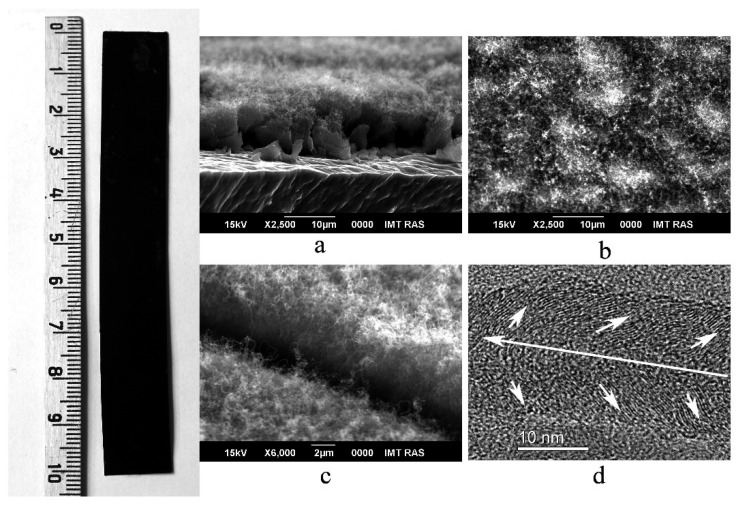
Left: a photograph of an MWCNT/Al foil strip; scanning electron micrographs of MWCNT/Al foil: (**a**) cross section, (**b**) top view, (**c**) a 45° view, and (**d**) transmission electron micrograph of individual MWCNT.

As reported in a previous article [31], the optimal conditions for the preparation of MWCNT/Al foil composites are 15–20 h of processing aluminum foil in a 20 wt% aqueous solution of Ni(NO_3_)_2_ at room temperature and deposition of MWCNT layer by pyrolysis of alcohol vapor at 600 °C within 1 h. Under these conditions, a continuous homogeneous layer with an average surface mass of MWCNTs (mass per surface area) of 0.3–0.4 mg/cm^2^ was deposited on the surface of the aluminum foil. Such MWCNT/Al samples were used in this work to increase their specific capacity by electrochemical oxidation. A photograph of an MWCNT/Al strip and typical scanning electron micrographs of the MWCNT layers are shown in Figure 1. The photograph of MWCNT/Al strip (left panel, Figure 1) clearly showed that the nanotube layer completely covered the substrate without any open spots. The scanning electron micrographs (Figure 1a–c) show a dense filamentary layer approximately 10 µm thick on the substrate. These results confirmed the fibrous nature of the deposit. The MWCNTs have a sinuous shape and are entangled, which indicates their high defectiveness [7]. The transmission electron micrograph in Figure 1d shows high resolution image of individual MWCNT. The MWCNT structure consists of many sp^2^-carbon basal planes (0001) which are visible in the tube image (Figure 1d) as a series of parallel lines with an average spacing of 0.36 nm. This value corresponds to the distance between the sp^2^-carbon basal planes in graphite-like structures [25]. The long and short arrows in Figure 1d show the directions of the tube axis and sp^2^-carbon planes, respectively. It is seen that the sp^2^-carbon planes are directed at an angle relative to the nanotube axis. Thus, the edges of the planes come out onto the surface of the nanotube, creating a large number of defects. (In perfect nanotubes, the sp^2^-carbon planes are directed along the axis; therefore, the surface of such tubes is rather inert.) The average diameter of MWCNTs is 20–50 nm. The grown MWCNT layers have excellent adhesion to the aluminum substrate, which allows them to be repeatedly twisted, bent, and also cut into pieces of the required size and shape. No delamination of the MWCNT layer from the substrate was observed. 

Electrochemical measurements of a two-electrode cell demonstrated the applicability of the MWCNT/Al foil as EDLC [31]. The average specific capacity of the MWCNTs was 30–40 F/g. However, in some samples, this value reached 60 F/g [31]. As noted in Introduction, such values are typical for non-modified carbon nanotubes. As will be shown below, the electrochemical oxidation of MWCNTs can significantly improve these characteristics. 

No information in the literature on the electrochemical oxidation of CNTs directly deposited on aluminum foil was found. The electrochemical oxidation of nanotubes in acidic and alkaline electrolytes has previously been reported [26,28,29]. In the work of Senokos et al. [27], a neutral aqueous Na_2_SO_4_ solution was used as an electrolyte. As the electrochemical oxidation of the MWCNTs grown on aluminum foil was done for the first time, the issue of choosing of suitable electrolyte was very critical. It was clear that acidic and alkaline electrolytes were not suitable due to the possible reaction with aluminum. In previous work [31], Na_2_SO_4_ solutions were used to measure the electrochemical characteristics of MWCNT/Al electrodes. The material has shown excellent resistance to this electrolyte. Therefore, in the present work, this electrolyte was chosen for experiments on the electrochemical oxidation of MWCNT/Al in order to increase their specific capacity. Preliminary experiments showed that during anodic oxidation of MWCNT/Al samples in aqueous solution of Na_2_SO_4_, the aluminum substrate was not etched if there is no open Al surface on it (fresh cuts). Thus, the aluminum foil coated with a layer of MWCNTs proved to be resistant to anodic etching, which allowed us to study in detail the effect of anodic oxidation of nanotubes on their electrochemical characteristics. 

**Figure 2:**
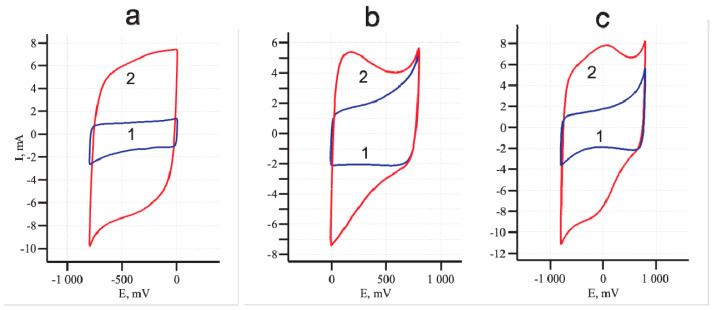
CVs of the MWCNT/Al foil in the three-electrode cell. Before electrochemical modification (1) and after anodic oxidation at 5 V for 20 min in 0.005 M aqueous Na_2_SO_4_ solution (2). Voltage scanning range: (**a**) From −800 to 10 mV; (**b**) From −10 to 800 mV; (**c**) From −800 to 800 mV.

Figure 2 shows an example of cyclic voltammograms of untreated and electrochemically oxidized MWCNT/Al samples. In contrast to the two-electrode configuration, in a three-electrode cell, the values of the specific capacitance and the shape of the cyclic voltammetric loops differ depending on the scanning range, as different ions are involved in the formation of an electric double layer in the cathodic and anodic ranges, leading to different redox processes. Traces 1 and 2 in Figure 2 correspond to cyclic voltammograms of the MWCNT/Al sample before and after oxidation. Oxidation led to an increase in the areas inside the voltammetric loops, which indicated an increase in the sample capacity. Figure 2a,b shows cyclic voltammograms in the cathodic and anodic ranges, respectively. As can be seen, in the separate scan windows, the effect of electrochemical oxidation was different. The general form of the cyclic voltammogram also changed after oxidation (Figure 2c). In particular, curve 2 in Figure 2b and curve 2 in Figure 2c showed the features of the peaks that could be associated with electrochemical redox reactions at the electrode. 

**Figure 3:**
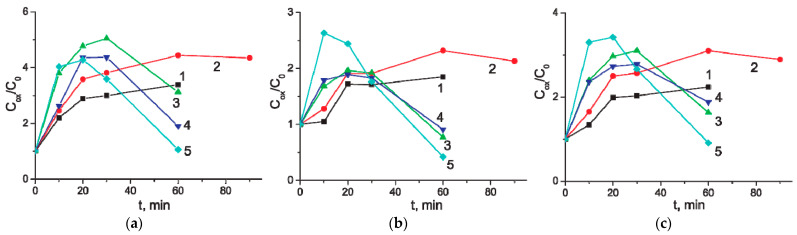
The ratio of the specific capacity of the modified and initial samples of MWCNT/Al (*C_ox_*/*C_0_*) versus the time of anodic oxidation. Electrolyte is 0.005 M aqueous Na_2_SO_4_ solution. Potential of the electrochemical oxidation: 1–3 V; 2–4 V; 3–5 V; 4–6 V; 5–7 V. CVs range: (**a**) From −800 to 10 mV; (**b**) From −10 to 800 mV; (**c**) From −800 to 800 mV.

As noted earlier, the MWCNT layer in our samples has excellent adhesion to the aluminum substrate. Handling them (cutting, bending, twisting, washing in water and electrolyte, etc.) did not lead to the exfoliation of the MWCNT layer. Therefore, after measurements in the electrochemical cell, the MWCNT/Al electrode can be washed with distilled water, dried, and used in the next experiment. No exfoliation of the carbon layer from the aluminum substrate occurs. The experiments with randomly chosen samples showed that the capacitive characteristics of the electrode are retained not only the next day, but also after weeks and even months of storage in air. The sample capacity changed after 6 months by ~10%. Due to this, we were able to study in sufficient detail the change in the capacitance characteristics of MWCNTs in the process of electrochemical oxidation by means of intermediate measurements at certain time intervals. Figure 3 shows the dependences of the increase in the specific capacity of MWCNTs on the time of electrochemical oxidation at different potentials. *C*_0_ is the initial capacity of the untreated MWCNT/Al electrode, calculated by the equation (1). It is known that the area inside the CV loop is proportional to the electric double layer capacitance of the electrode. A standard approach was used to measure the specific capacity of MWCNTs on the electrode [23,25]. The cell capacity was calculated by Equation (1):

***C_cell_*** = **∫*IdV***/(**∆*V* • *ν***) (1)

where ***C_cell_*** is the capacity of the cell; ***∫IdV*** is the area under the CV characteristic at I > 0 (V•A). (This is half of the area inside the complete loop in Figure 2); **∆*V*** is the voltage range (V); ***ν*** is the voltage scan rate (V/s). 

The capacity of the working electrode in a three-electrode cell is equal to the capacity of the cell. Therefore, the specific capacity of MWCNTs (***C_sp_***) is ***C_cell_/m_M__WCNT,_*** where ***m_M__WCNT_*** is the mass of MWCNTs on the electrode. 

*C_ox_* is the capacity of the sample after electrochemical oxidation. Thus, the *C_ox_/C_0_* ratio shows how much the electrode capacitance changed after the treatment. 

The MWCNT/Al electrodes were subjected to electrochemical oxidation at potentials from 3 to 7 V. At certain intervals, the capacitance of these samples was measured. The relative change in capacitance is shown in Figure 3. Thus, the traces on the figure show the dynamics of changes in the capacity of MWCNT/Al electrodes during the process of electrochemical oxidation under different conditions. As can be seen, the electrochemical oxidation of MWCNTs can lead to a multiple increase in their specific capacity. In 0.005 M aqueous Na_2_SO_4_ solution, the capacity reaches its maximum value within 20–30 min, after which it changes little for anodization potentials of 3–4 V. At higher oxidation potentials (5–7 V), treatment for more than 30 min leads to a decrease in capacity. Table 1 contains detailed data on the changes in the specific capacity of MWCNTs after anodic oxidation at various potential.

**Figure 4:**
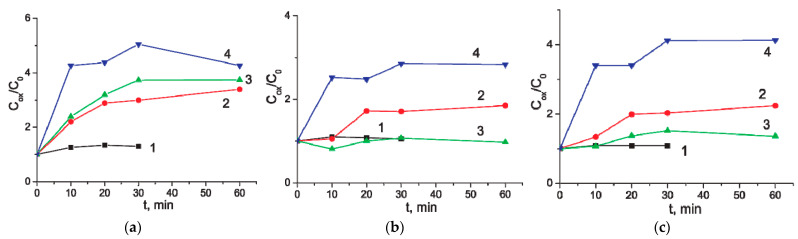
The *C_ox_*/*C_0_* ratio of MWCNT/Al versus the time of anodic oxidation. Potential of anodic oxidation was 3 V. Concentration of the electrolyte (aqueous Na_2_SO_4_ solution), mol/L: 0, i.e., distilled water (trace 1), 0.005 (trace 2), 0.05 (trace 3), and 0.5 (trace 4). CV range: (**a**) From −800 to 10 mV; (**b**) From −10 to 800 mV; (**c**) From −800 to 800 mV.

In addition to the anodizing potential, the electrolyte concentration plays a significant role in the process of electrochemical modification of MWCNTs. Figure 4 shows the time dependences of the increase in the specific capacity of MWCNTs during oxidation in a solution of Na_2_SO_4_ of different concentrations, as well as in distilled water. When processing in pure water, practically no changes in properties are observed (Figure 4, trace 1). With increasing salt concentration, the rate of growth in the specific capacity of MWCNTs increases. However, oxidation at a potential above 5 V in a 0.5 M Na_2_SO_4_ solution leads to the rapid destruction of the continuous MWCNT layer and the appearance of open areas of the substrate. More details on the change in the specific capacity of MWCNTs after anodic oxidation at various electrolyte concentrations are given in Table 2.

Thus, the optimal conditions for improving the capacitive characteristics of MWCNT/Al electrodes can be considered an oxidation potential in the range of 4–5 V, a moderate electrolyte concentration of 0.005–0.05 mol/L, and a processing time of 20–30 min. The electrodes treated in this way have a specific capacity 4–5 times higher than the original ones. At the same time, the integrity of the MWCNT layer and excellent adhesion to the Al substrate are retained. 

To suggest the main reasons for the increase in the specific capacity of MWCNTs after electrochemical oxidation, the features of the electrochemical behavior of the modified electrodes were investigated, and the materials were tested using methods that provide information on changes in the surface properties of MWCNTs.

**Figure 5:**
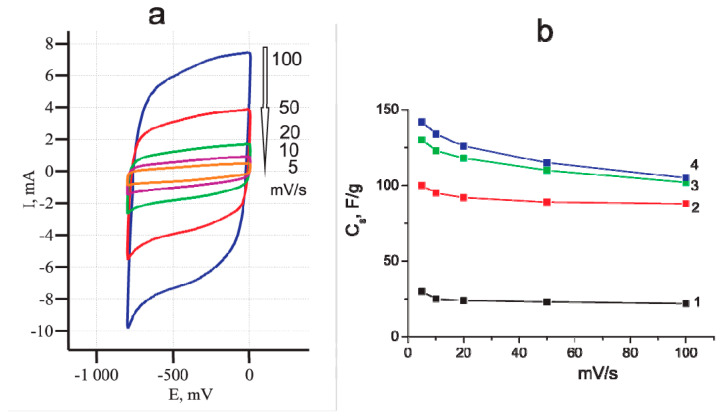
(**a**) CVs at the different scan rates. Working electrode was the MWCNT/Al foil oxidized at 5 V for 20 min; (**b**) the dependences of the specific capacity on the scanning rate. Oxidation time, min: 0 (trace 1); 10 (trace 2); 20 (trace 3); 30 (trace 4).

First, attention should be paid to the change in the cyclic voltammetric shape after oxidation of the MWCNT/Al electrode (Figure 2). The presence of maximum and minimum on the curve may indicate the occurrence of reversible electrochemical reactions with oxygen-containing functional groups [7]. Ideal EDLS has rectangular voltammetric characteristics. In this case, the accumulation of energy is purely electrostatic and does not depend on the potential. The sign of current is immediately reversed upon reversal of the potential sweep [7]. The deviation of the CV shape from rectangular in real supercapacitors is due to a number of reasons (ohmic resistance, difficulties in ion diffusion, redox processes). The CVs of the oxidized MWCNT/Al sample at different scanning rates (Figure 5a), had a shape close to rectangular, which confirmed the main mechanism of charge accumulation as an electric double layer. 

Figure 5b shows the dependences of the calculated specific capacity on the scanning speed for the MWCNT/Al sample with different oxidation degrees. They were obtained by intermediate measurements of the characteristics of one sample oxidized at a potential of 5 V in a 0.005 M Na_2_SO_4_ solution. In the general, the specific capacitance value depends on the CV scanning rate, as the formation of an electric double layer takes a certain time associated with the diffusion of ions to the electrode surface. In trace 1, obtained using the initial (nonoxidized) sample, the specific capacity does not increase much with decreasing scanning speed. This may indicate the absence of serious diffusion difficulties during the formation of the electric double layer [7,8,9]. As indicated by trace 2–4 in Figure 5b, as the sample was oxidized for duration from 10 min to 30 min, the specific capacity of MWCNTs increased by almost 5 times its original value. The character of the dependence of the ***C_sp_*** on the scanning rate remains the same. However, the relative increase in the specific capacity with a decrease in the scanning speed for the oxidized sample (~30%) is greater than that for the initial one (~15%) (Figure 5b). This observation can be explained by an increase in the specific surface area and porosity of MWCNTs due to electrochemical etching. 

**Figure 6:**
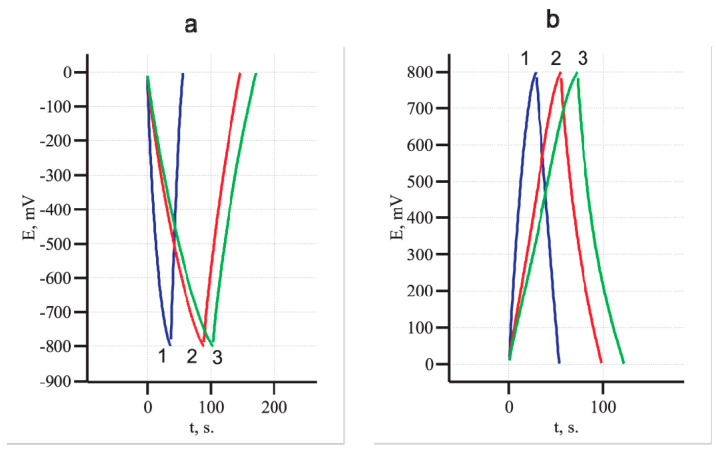
Charge–discharge curves of the MWCNT/Al sample; (**a**) cathodic and (**b**) anodic ranges. Oxidation time, min: 0 (trace 1); 10 (trace 2); 20 (trace 3).

Important data on the nature of the electrochemical processes occurring at the electrode can be obtained from the galvanostatic charge–discharge experiments. A triangular shape of the charge–discharge curves is characteristic of the EDLC, and the slope of the discharge curve is proportional to the electrode capacitance. Figure 6 presents the charge-discharge curves of one MWCNT/Al sample: pristine and subsequently oxidized at 5 V in 0.005 M Na_2_SO_4_ solution for 10 and 20 min. It can be seen that as oxidation proceeds, the slope of the discharge curve decreases, which indicates an increase in the sample capacity. At the same time, the shape of the charge–discharge dependences remains triangular in both the cathodic and anodic regions. This suggests that the accumulation of charge occurs due to the formation of an electric double layer. 

**Figure 7:**
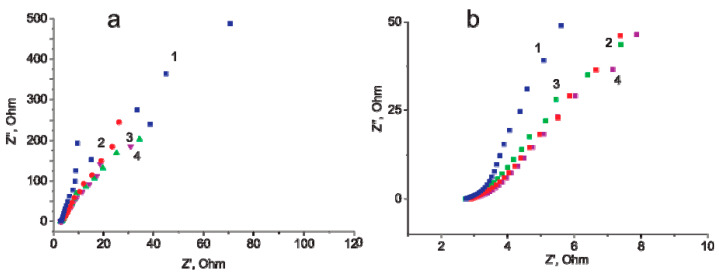
Nyquist plots of 0.5 M Na_2_SO_4_ solution at a MWCNT/Al electrode. Oxidation time: 0 min (trace 1), 10 min (trace 2), 20 min (trace 3) and 30 min (trace 4). (**a**) Full frequency range. (**b**) Middle frequency range.

The conclusions drawn from the experiments described above are also confirmed by the results of impedance spectroscopy. Figure 7 presents the Nyquist (imaginary impedance (Z”) versus real impedance (Z’)) plots of 0.5 M Na_2_SO_4_ solution at the MWCNT/Al electrode. In the experiments, a pristine MWCNT/Al electrode oxidized at 5 V in a 0.005 M Na_2_SO_4_ solution for 10, 20 and 30 min was used. One of the simplest ways to describe the supercapacitor frequency behavior is to associate a serial resistance and a capacitance [34]. In the Nyquist plot, the ohmic resistance defines the high-frequency region (bottom of the plot) and the capacitance defines the low-frequency region (top of the plot). For ideal EDLS electrode (for example, polished glassy carbon), the Nyquist plot is a semi-infinite straight line parallel to the ordinate axis (Z”). However, for real electrodes there is a deviation of the Nyquist plot from an angle of 90°. This may be due to the inhomogeneity of the electrode surface.

As can be seen from Figure 7a, the shape of the Nyquist plot is near linear for the MWCNT/Al sample before oxidation and remains so after its oxidation. This is typical of supercapacitors. In a Nyquist plot, the ohmic resistance of a cell is estimated by the offset of Z’ on the abscissa in the high frequency region. Accordingly, the ohmic resistance was ~3 Ω for both the non-oxidized and oxidized electrode. These results indicate that oxidation did not lead to an increase in the ohmic resistance of the cell. At the same time, the slope of the Nyquist plot for the oxidized sample decreases with increasing oxidation time. Figure 7b clearly shows this trend. This behavior can be explained by an increase in the porosity of MWCNTs as a result of electrochemical oxidation [34].

**Figure 8:**
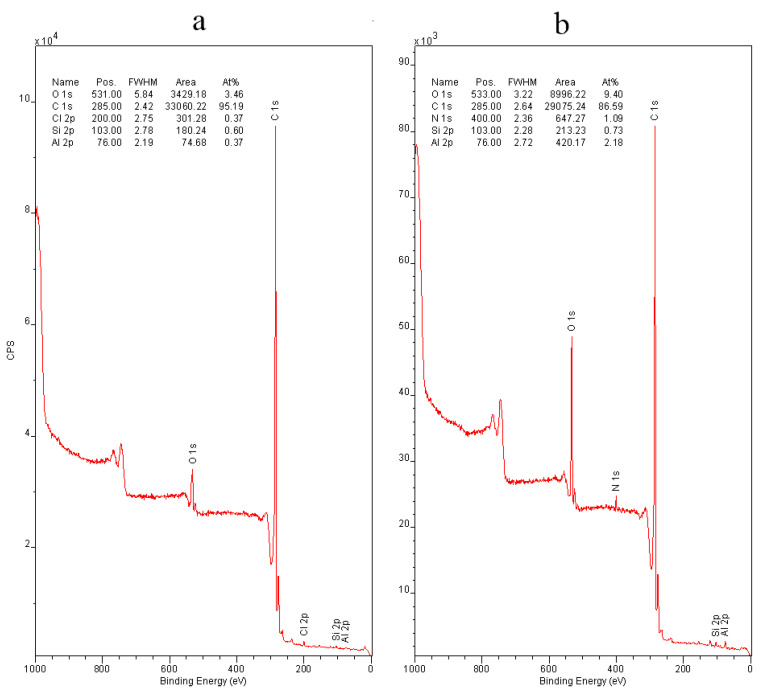
Survey X-ray photoelectron spectra. (**a**) Initial MWCNTs/Al sample. (**b**) Oxidized MWCNTs/Al sample.

Additional information on changes in MWCNTs after electrochemical oxidation was obtained from the X-ray photoelectron spectra of the samples. X-ray photoelectron spectroscopy is a semi-quantitative method for investigation the elemental composition, chemical and electronic state of atoms on the surface of the material. A comparison was made of the spectra of the initial MWCNT/Al sample and the sample oxidized at 5 V in a 0.005 M Na_2_SO_4_ solution for 20 min. As can be seen from the survey spectrum (Figure 8), surfaces both initial (Figure 8a) and oxidized MWCNTs contain mainly carbon and oxygen atoms. Based on the areas of the O 1 s peaks at 600 eV in the survey spectrum, the total concentration of surface oxygen increased almost 3 times (up to 9.4 atomic %) in the oxidized sample compared to the initial. 

**Figure 9:**
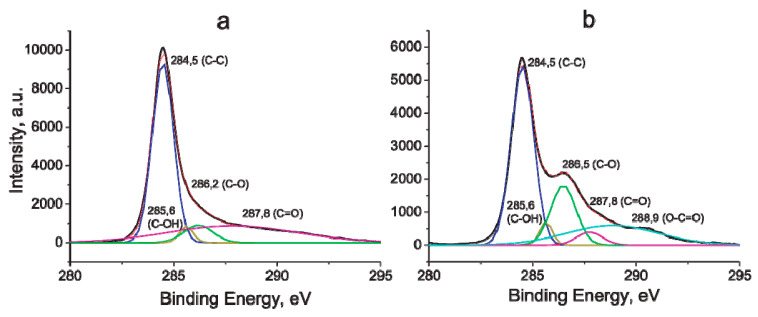
High-resolution carbon XPS spectra. (**a**) Initial MWCNT/Al sample. (**b**) Oxidized MWCNT/Al sample.

Characteristic changes are observed in the high-resolution carbon X-ray photoelectron spectra (Figure 9). In the spectra of both samples, bands related to oxygen-containing functional groups [15] can be observed. However, an additional maximum at 286.5 eV in the spectrum of the oxidized sample indicates that the concentration of oxygen-containing groups on its surface is higher than on the surface of the initial sample (Figure 9). According to the peak-fitted results, an increase in the concentration of such groups as -CO, -C=O, O-C=O is observed, which is consistent with the literature data [15,26,28].

**Figure 10:**
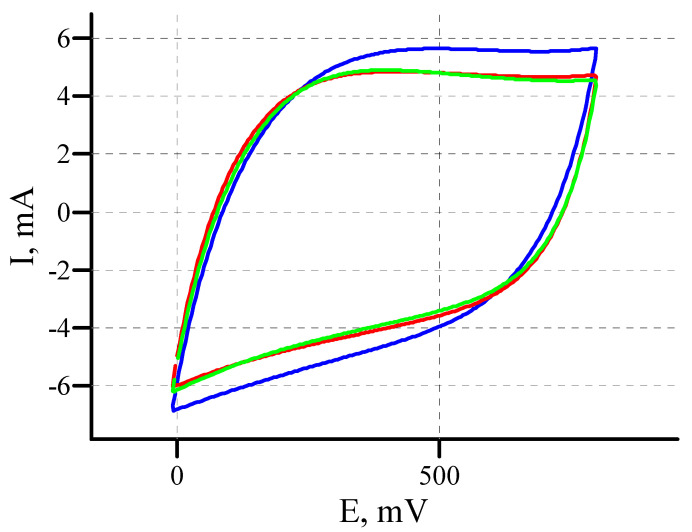
Multiple CVs of the oxidized MWCNT/Al sample. Cycle number: 1—blue, 10,000—red, 20,000—green.

One of the main advantages of supercapacitors is their resistance to multiple charge-discharge cycles. In the case of oxidized nanotubes, it is especially important to check how the acquired improved characteristics will be retained under long-term operating conditions. We tested a MWCNT/Al sample oxidized at 5 V for 20 min. The test included 20,000 cyclic voltammograms in the anodic and cathodic ranges at a scan rate of 1000 mV/s. The sample demonstrated excellent stability. Figure 10 shows the cyclic voltammograms in the anode range with 1st, 10,000th and 20,000th scan. It can be seen that after a slight decrease in the capacity at the beginning of cycling (~5%), the characteristics of the sample remain unchanged in the future. It is also important that the cyclic voltammetric shape remains unchanged, which indicates the absence of degradation of the active layer.

The study showed that during the anodic oxidation of MWCNTs deposited on aluminum foil, certain changes occurred. First, there is a functionalization of MWCNTs, which consists in a significant increase in oxygen-containing groups covalently bonded to the surface of the tubes. The results of electrochemical tests indirectly indicate an increase in the porosity of MWCNTs. These results are generally consistent with those reported in the literature [26,27,28,29]. Several reasons for the increase in the specific capacity of MWCNTs after oxidative modification are discussed in the literature. On the one hand, an increase in capacity can occur due to redox processes with the participation of oxygen-containing groups, which are formed on the surface of carbon nanotubes during oxidation. Another reason is the increase in porosity and specific surface area of MWCNTs, which is the result of electrochemical etching of the surface of nanotubes. According to the work of Ye et al. [29], an increase in capacity could be attributed to the increasing hydrophilic characteristics of nanotubes. In our research, we also found an increase in the concentration of oxygen-containing groups on the surface of oxidized nanotubes. A change in the cyclic voltammetric shape may indicate the participation of functional groups in the process of energy accumulation. However, it is impossible to explain the 4–5-fold increase in specific capacity only by redox processes. In general, as the studies have shown, oxidized MWCNT/Al electrodes retain the charge accumulation mechanism due to the formation of an electric double layer. Most likely, the reason for the strong increase in capacity as result of the electrochemical oxidation of MWCNT/Al electrodes is due to a combination of several factors mentioned above.

## 4. Conclusions

An original method for growing MWCNT layers on aluminum foil was used to develop electrodes for EDLCs. It is shown that a simple operation of electrochemical oxidation of MWCNT/Al foil samples makes it possible to increase the specific capacity by a factor of 4–5. An aqueous solution of sodium sulfate was used as the electrolyte. The best results were obtained upon oxidation of MWCNT/Al samples for 20–30 min at a potential of 4–5 V in an electrolyte with a concentration of 0.005 mol/L. The specific capacity of MWCNTs after modification reached 140 F/g. The improved characteristics of oxidized samples are retained with multiple charge–discharge cycles (up to 20,000). The samples do not degrade when stored for a long time in an ambient atmosphere (up to 6 months). The results obtained significantly expand the possibilities of using MWCNTs grown on Al foil as electrodes for supercapacitors.

## Figures and Tables

**Table 1:** The *C_ox_*/*C_0_* ratio of MWCNT/Al for various time of anodic oxidation at different potential. Electrolyte was 0.005 M aqueous Na_2_SO_4_ solution.

Potential of the Oxidation, V	CV Range, mV	10 min	20 min	30 min	60 min	90 min
3	−800 to 10	2.2	2.9	3.0	3.4	-
	−10 to 800	1.1	1.7	1.7	1.9	-
	−800 to 800	1.3	2.0	2.0	2.2	-
4	−800 to 10	2.5	3.6	3.8	4.4	4.4
	−10 to 800	1.3	1.9	1.9	2.3	2.1
	−800 to 800	1.7	2.5	2.6	3.1	2.9
5	−800 to 10	3.8	4.8	5.0	3.1	-
	−10 to 800	1.7	2.0	1.9	0.8	-
	−800 to 800	2.0	3.0	3.1	1.6	-
6	−800 to 10	2.6	4.4	4.4	1.9	-
	−10 to 800	3.8	1.9	1.8	0.9	-
	−800 to 800	1.4	2.7	2.8	1.8	-
7	−800 to 10	4.0	4.3	3.6	1.1	-
	−10 to 800	2.6	2.4	2.8	0.4	-
	−800 to 800	3.3	3.4	1.7	0.9	-

**Table 2:** The *C_ox_*/*C_0_* ratio of MWCNT/Al for various time of anodic oxidation in the electrolyte of different concentration (aqueous Na_2_SO_4_ solution). The potential of anodic oxidation was 3 V.

Concentration of Na_2_SO_4_ Solution, mol/L	CV Range, mV	10 min	20 min	30 min	60 min
0	−800 to 10	1.3	1.3	1.3	-
	−10 to 800	1.1	1.1	1.1	-
	−800 to 800	1.1	1.1	1.1	-
0.005	−800 to 10	2.2	2.9	3.0	3.4
	−10 to 800	1.1	1.7	1.7	1.9
	−800 to 800	1.3	2.0	2.0	2.2
0.05	−800 to 10	2.4	3.2	3.7	3.7
	−10 to 800	0.8	1.0	1.1	1.0
	−800 to 800	1.1	1,4	1.5	1.5
0.5	−800 to 10	4.3	4.4	5.1	4.3
	−10 to 800	2.5	2.5	2.9	2.8
	−800 to 800	3.4	3.4	4.1	4.1
